# A comparative study on the features of breast sclerosing adenosis and invasive ductal carcinoma via ultrasound and establishment of a predictive nomogram

**DOI:** 10.3389/fonc.2023.1276524

**Published:** 2023-10-23

**Authors:** Yuan Li, Xiu-liang Wei, Kun-kun Pang, Ping-juan Ni, Mei Wu, Juan Xiao, Lu-lu Zhang, Fei-xue Zhang

**Affiliations:** ^1^ Department of Ultrasound, the Second Hospital, Cheeloo College of Medicine, Shandong University, Jinan, Shandong, China; ^2^ Center of Evidence-Based Medicine, Institute of Medical Sciences, the Second Hospital, Cheeloo College of Medicine, Shandong University, Jinan, Shandong, China; ^3^ Department of Pathology, the Second Hospital, Cheeloo College of Medicine, Shandong University, Jinan, Shandong, China

**Keywords:** sclerosing adenosis, invasive ductal carcinoma, ultrasonic features, BI-RADS, nomogram

## Abstract

**Objective:**

To analyze the clinical and ultrasonic characteristics of breast sclerosing adenosis (SA) and invasive ductal carcinoma (IDC), and construct a predictive nomogram for SA.

**Materials and methods:**

A total of 865 patients were recruited at the Second Hospital of Shandong University from January 2016 to November 2022. All patients underwent routine breast ultrasound examinations before surgery, and the diagnosis was confirmed by histopathological examination following the operation. Ultrasonic features were recorded using the Breast Imaging Data and Reporting System (BI-RADS). Of the 865 patients, 203 (252 nodules) were diagnosed as SA and 662 (731 nodules) as IDC. They were randomly divided into a training set and a validation set at a ratio of 6:4. Lastly, the difference in clinical characteristics and ultrasonic features were comparatively analyzed.

**Result:**

There was a statistically significant difference in multiple clinical and ultrasonic features between SA and IDC (*P*<0.05). As age and lesion size increased, the probability of SA significantly decreased, with a cut-off value of 36 years old and 10 mm, respectively. In the logistic regression analysis of the training set, age, nodule size, menopausal status, clinical symptoms, palpability of lesions, margins, internal echo, color Doppler flow imaging (CDFI) grading, and resistance index (RI) were statistically significant (*P*<0.05). These indicators were included in the static and dynamic nomogram model, which showed high predictive performance, calibration and clinical value in both the training and validation sets.

**Conclusion:**

SA should be suspected in asymptomatic young women, especially those younger than 36 years of age, who present with small-size lesions (especially less than 10 mm) with distinct margins, homogeneous internal echo, and lack of blood supply. The nomogram model can provide a more convenient tool for clinicians.

## 1 Introduction

Sclerosing adenosis (SA) is a benign proliferative breast disease. The incidence rate of benign breast lesions in biopsy can reach up to 30%. Interestingly, SA may coexist with other proliferative diseases or malignancies. Although not considered a premalignant lesion, SA is associated with an increased risk of breast cancer (1–3). It is typically small and asymptomatic and may be difficult to palpate or fix in position during physical examination (4). Pathologically, SA is characterized by the proliferation of acini and fibrous connective tissue that distorts the normal architecture of the lobules. These features lead to SA manifesting an invasive appearance that is challenging to differentiate from breast carcinoma clinically and radiologically. Therefore, histopathological examination remains the gold standard for the diagnosis of SA (5, 6).

Ultrasonography is an important diagnostic and screening method for breast diseases. It is less affected by gland density (7). Currently, the overall sensitivity of ultrasound in diagnosing breast cancer is 72.2%-86.3% (8). Among all breast cancers, invasive ductal carcinoma (IDC) is the most common pathological type of breast cancer, accounting for roughly 80% of all breast cancers (9). However, owing to the pathological characteristics of SA, its ultrasound features mimic malignancies, such as calcification, blurred edges, and abundant blood flow, resulting in a false positive rate of up to 45% in the ultrasonic diagnosis of SA (10). IDC necessitates surgical resection, whereas SA can be monitored by imaging techniques (11). Therefore, there is an urgent need to differentiate between SA and IDC preoperatively.

Nomograms are widely used to predict disease prognosis and medical event outcomes by combining multiple risk factors (12). This study comparatively analyzed the clinical and ultrasonic characteristics of SA and IDC, clarified the characteristic manifestations of SA, and constructed a predictive nomogram for SA. Additionally, we aimed to enhance the ultrasound diagnosis rate of SA.

## 2 Materials and methods

### 2.1 Participants and study design

The medical records and imaging data of patients with SA and IDC diagnosed via histopathological examination from January 2016 to November 2022 at the Second Hospital of Shandong University were collected, comprising 203 female patients (252 nodules) suffering from SA and 662 IDC female patients (731 nodules). All patients underwent routine breast ultrasound examinations before surgery. The diagnosis was confirmed by histopathological examination after surgery. The hematoxylin and eosin (H&E) staining was used for histological examination following a routine procedure. The clinical information was obtained from the inpatient electronic medical record system, whilst ultrasound images were gathered from the ultrasound workstation.

**Figure 1:**
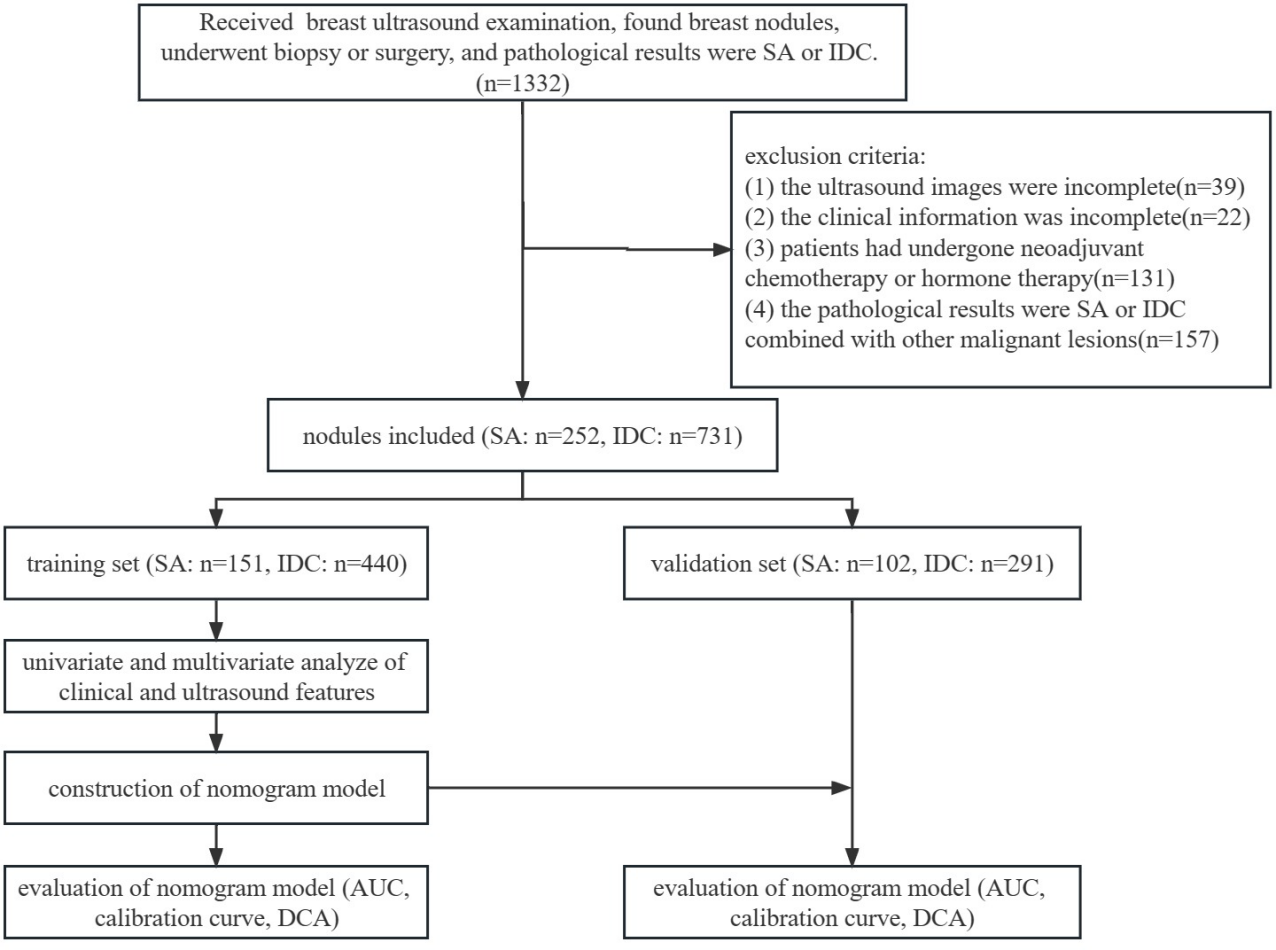
Flowchart of this study.

The inclusion criteria were as follows: (1) ultrasonic examination was performed at the Second Hospital of Shandong University; (2) all nodules were pathologically confirmed as SA or IDC combined with other benign lesions after biopsy or surgery; (3) available ultrasound images and clinical information. The exclusion criteria were as follows: (1) incomplete ultrasound images or clinical information; (2) patients had previously undergone neoadjuvant chemotherapy or hormone therapy; (3) the pathological results were SA or IDC combined with other malignant lesions. The patients were randomly divided into a training set (SA: 120 patients, 151 nodules; IDC: 399 patients, 440 nodules) and a validation set (SA: 83 patients, 102 nodules; IDC: 263 patients, 291 nodules) at a ratio of 6:4. The flowchart of this study is illustrated in **Figure 1**.

### 2.2 Instruments and methods

The LOGIQ E9 color ultrasonic diagnostic apparatus (GE Healthcare, Wauwatosa, WI) with linear array probe (9–15 MHz frequency) was used. The patient was placed in the supine position with the bilateral mammary glands and armpits exposed. Then, a scan was performed on the radial planes around the nipple in a clockwise or counterclockwise direction, until the breast and armpit scans in all quadrants were completed. Subsequently, the lesions were scanned and thoroughly visualized in multiple sections. The nodules’ long diameter and thick diameter were measured on the maximum diametral section, while the transverse diameter was measured on the vertical section. Following the BI-RADS standard, sections containing significantultrasonic characteristics of nodules were retained, and the characteristics of nodules were described and recorded. The research contents chiefly included 12 items: size, shape, margin, echo, internal echo, aspect ratio, posterior feature, calcification, echogenic rim, color Doppler flow imaging (CDFI) grading, and resistance index (RI) of the nodule. The maximum diameter of the nodule was labeled as the size of the lesion.

### 2.3 Ultrasound evaluation

All examinations were performed by sonographers who had been practiced breast ultrasound for more than 3 years. All images were retrospectively analyzed by two sonographers with 14 years and 10 years of experience in breast ultrasound diagnosis, respectively.They were blinded to the pathological results of the nodules. The Adler semi-quantitative method CDFI was used to classify the blood flow signal of the nodule into four levels (13): level 0: no blood flow within the nodule; level I: minimal blood flow, with 1-2 rod-shaped and punctate blood flow within the nodule; level II: moderate blood flow, with 1 long or 3-4 punctate blood vessels within the nodule; level III: rich blood flow, with 2 long or more than 5 punctate blood vessels within the nodule. All nodules were classified according to the Breast Imaging Data and Reporting System (BI-RADS) standard. Although grade 4a nodules have a low malignancy risk, a biopsy is frequently required in clinical practice to determine the nature of the nodules (14–16). Therefore, in our study, nodules of BI-RADS 3 were classified as benign, whereas nodules of BI-RADS 4a, 4b, 4c, and 5 were categorized as malignant. Disagreements between the two physicians on the same nodule were resolved by reaching a consensus.

### 2.4 Statistical analysis

SPSS 21.0 (IBM Corporation, Armonk, NY) statistical software was employed for statistical analysis. Continuous variables following normal distribution were evaluated using the Kolmogorov-Smirnov test. Data following normal distribution were expressed as mean ± standard deviation (SD) and analyzed by Student’s t-test. Data not following normal distribution were expressed by the median, 25th percentile (P25), and 75th percentile (P75) and analyzed by Mann–Whitney U test. Categorical variables were presented as numbers and percentages and analyzed by χ^2^ test and Fisher's exact test. Multiple factor analysis was conducted using logistic regression analysis. The R software (version 4.2.3) was used to construct and evaluate the nomogram model. The receiver operating characteristic (ROC) curve was plotted to evaluate the model’s predictive ability using the area under the curve (AUC) and 95% confidence intervals (CI). A calibration curve was constructed to assess the calibration of the model. Decision curve analysis (DCA) was utilized to examine clinical benefits. Restricted cubic splines (RCS) were used to analyze the nonlinear relationship between continuous variables and the likelihood of SA. The online dynamic nomogram was built with Shiny. *P*<0.05 was considered statistically significant.

This study was approved by the ethical review board of the Second Hospital of Shandong University (KYLL-2023LW042). Informed consent was obtained from all patients. All experiments were performed in accordance with the Declaration of Helsinki and the relevant guidelines.

## 3 Results

### 3.1 Clinical characteristics in the training and validation sets

There were significant differences in the clinical data between SA and IDC patients in the training and validation sets in terms of age, menopausal status, clinical symptoms (including conscious breast lumps, pain, nipple discharge, breast skin changes, etc.), palpation, and the number of lesions (single or multiple) (*P*<0.05). The summary of patient clinical characteristics is detailed in **Table 1**.

**Table 1:** Univariate analysis of clinical characteristics in training and validation sets.

	Training set		*T/U/χ^2^*	*P*	Validation set		*t/χ^2^*	*P*
	SA*	IDC †			SA	IDC		
Age(year)^‡^	40.0 ± 12.3	53.6 ± 12.2	11.155	**<0.001§**	42.2 ± 10.8	52.3 ± 11.4	7.097	**<0.001**
Family history of breast cancer			0.344	0.506			0.299	0.793
Yes	8(6.7%)	21(5.3%)			4(4.8%)	17(6.5%)		
No	112(93.3%)	378(94.7%)			79(95.2%)	246(93.5%)		
Menopausal state			83.305	**<0.001**			33.271	**<0.001**
Premenopausal	106(88.3%)	163(40.9%)			67(80.7%)	117(44.5%)		
Postmenopausal	14(11.7%)	236(59.1%)			16(19.3%)	146(55.5%)		
Clinical symptoms			42.289	**<0.001**			32.575	**<0.001**
Symptomatic	65(54.2%)	331(83.0%)			46(55.4%)	224(85.2%)		
Asymptomatic	55(45.8%)	68(17.0%)			37(44.6%)	39(14.8%)		
Palpation			99.191	**<0.001**			34.755	**<0.001**
Palpable	83(55.0%)	401(91.1%)			68(66.7%)	265(91.1%)		
Unpalpable	68(45.0%)	39(8.9%)			34(33.3%)	26(8.9%)		
Number of lesions			5.385	**0.033**			9.221	**0.006**
Simple	99(82.5%)	360(90.2%)			67(80.7%)	243(92.4%)		
Multiple	21(17.5%)	39(9.8%)			16(19.3%)	20(7.6%)		

^*^sclerosing adenosis; ^†^invasive ductal carcinoma; ^‡^data with normal distribution are shown by the mean ± standard deviations. § The bold values indicate that the P-values have statistical significance (P<0.05).

### 3.2 Ultrasonic characteristics in the training and validation sets

In the training set, significant differences were noted between SA and IDC (*P*<0.001) in 10 ultrasound features, including size, shape, margin, echo, internal echo, aspect ratio, posterior feature, calcification, CDFI grading, and RI. In the validation set, significant differences were observed between SA and IDC (*P*<0.001) in 11 ultrasound features, comprising size, shape, margin, echo, internal echo, aspect ratio, posterior feature, calcification, echogenic rim, CDFI grading, and RI. In the training and validation sets, 42.4% and 48.0% of the SA group were classified as BI-RADS 4a or above, respectively, while only 3.0% and 3.1% were classified as BI-RADS 3 in the IDC group. As anticipated, the rate of misdiagnosis of SA was significantly higher than that of IDC. The specific analytical results of ultrasound characteristics are summarized in **Table 2**.

**Table 2:** Univariate analysis of ultrasonic features in training and validation sets.

	Training set		*T/U/χ^2^*	*P*	Validation set		*T/U/χ^2^*	*P*
	SA* (n=151)	IDC† (n=440)			SA (n=102)	IDC (n=291)		
Size (mm)**	11.00(7.00, 16.00)	20.00(14.00, 29.00)	11.065	**<0.001††**	11.00(8.00, 17.00)	22.00(15.00, 29.00)	8.539	**<0.001**
Shape			146.650	**<0.001**			142.584	**<0.001**
Regular	88(58.3%)	46(10.5%)			63(61.8%)	18(6.2%)		
Irregular	63(41.7%)	394(89.5%)			39(38.2%)	273(93.8%)		
Margin			139.342	**<0.001**			110.264	**<0.001**
Distinct	107(70.9%)	83(18.9%)			71(69.6%)	43(14.8%)		
Indistinct	44(29.1%)	357(81.1%)			31(30.4%)	248(85.2%)		
Internal echo			128.718	**<0.001**			95.789	**<0.001**
Homogeneity	49(32.5%)	6(1.4%)			35(34.3%)	3(1.0%)		
Heterogeneity	102(67.5%)	434(98.6%)			67(65.7%)	288(99.0%)		
Echo pattern			14.435	**0.001**			16.881	**<0.001**
Hyper/isoechoic	3(2.0%)	4(0.9%)			1(1.0%)	5(1.7%)		
Hypoechoic	130(86.1%)	419(95.2%)			88(86.3%)	279(95.9%)		
Complex cystic and solid	18(11.9%)	17(3.9%)			13(12.7%)	7(2.4%)		
Aspect ratio			4.274	**0.039**			4.091	**0.048**
<1	135(89.4%)	362(82.3%)			91(89.2%)	234(80.4%)		
≥1	16(10.6%)	78(17.7%)			11(10.8%)	57(19.6%)		
Posterior echo			31.213	**<0.001**			17.823	**<0.001**
Unchanged	92(60.9%)	201(45.7%)			67(65.7%)	137(47.1%)		
Enhanced	30(19.9%)	46(10.5%)			16(15.7%)	32(11.0%)		
Shadow	29(19.2%)	193(43.8%)			19(18.6%)	122(41.9%)		
Calcification			54.166	**<0.001**			36.176	**<0.001**
None	124(82.1%)	220(50.0%)			82(80.4%)	159(54.6%)		
Microcalcification or punched	19(12.6%)	203(46.1%)			10(9.8%)	121(41.6%)		
macrocalcification	8(5.3%)	17(3.9%)			10(9.8%)	11(3.8%)		
Echogenic rim			3.152	0.076			5.805	**0.015**
Absent	137(90.7%)	374(85.0%)			96(94.1%)	247(84.9%)		
Present	14(9.3%)	66(15.0%)			6(5.9%)	44(15.1%)		
CDFI^‡^ grading			60.417	**<0.001**			38.254	**<0.001**
Level 0-I	146(96.7%)	281(63.9%)			100(98.0%)	196(67.4%)		
Level II-III	5(3.3%)	159(36.1%)			2(2.0%)	95(32.6%)		
RI^§^			174.012	**<0.001**			82.743	**<0.001**
≥0.70	16(10.6%)	318(72.3%)			19(18.6%)	205(70.4%)		
<0.70	135(89.4%)	122(27.7%)			83(81.4%)	86(29.6%)		
BI-RADS ^||^			238.944	**<0.001**			135.742	**<0.001**
≤3	87(57.6%)	13(3.0%)			53(52.0%)	9(3.1%)		
≥4a	64(42.4%)	427(97.0%)			49(48.0%)	282(96.9%)		

^*^sclerosing adenosis; ^†^invasive ductal carcinoma; ^‡^Color Doppler Flow Imaging; ^§^resistance index; ^||^Breast Imaging Data and Reporting System; ** data with the non-normal distribution are shown by the median (P25, P75). †† The bold values indicate that the P-values have statistical significance (P<0.05).

### 3.3 Multivariate logistic regression analysis in the training set and construction of nomogram

Statistically significant variables from the single-factor analysis of the training set were incorporated into the logistic model (χ2 = 366.874, *P*<0.001). Among the independent variables inputted in the model, differences in age, size, menopausal status, clinical symptoms, palpation, margin, internal echo, CDFI grading, and RI were statistically significant (*P*<0.05) (**Table 3**).

**Table 3:** Multivariate logistic regression analysis in the training set.

	β	P	OR^*^	OR 95%CI^†^	
				Lower limit	Upper limit
Age (year)	3.254	0.000	25.892	4.292	156.213
Size (mm)	1.445	0.000	4.242	1.883	9.560
Premenopausal	-1.440	0.013	0.237	0.076	0.734
Asymptomatic	-1.175	0.008	0.309	0.129	0.738
Unpalpable	-1.101	0.037	0.333	0.118	0.937
Distinct margin	-1.386	0.001	0.250	0.107	0.585
Homogeneity	-2.239	0.002	0.107	0.026	0.439
CDFI^‡^ grading level 0-I	-1.599	0.022	0.202	0.051	0.798
RI^§^<0.70	-1.183	0.010	0.306	0.124	0.755

^*^odds ratio; ^†^confidence internal; ^‡^ Color Doppler Flow Imaging; ^§^resistance index.

**Figure 2:**
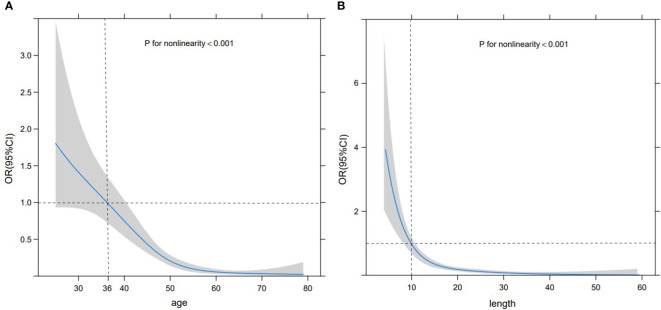
RCS curve. RCS curve displaying the relationship between age **(A)** and size **(B)** with the probability of SA. The gray shaded area represents 95% CI, the black horizontal dashed line represents OR=1, and the black vertical dashed line denotes the cut-off values of age and size.

Based on the RCS curve trend, the corresponding abscissa value when odds ratio (OR) =1 was selected as the cut-off value. As the patient age increased, the likelihood of SA significantly decreased, with a cut-off value of 36 years old and stabilizing after 55 years old. At the same time, as the size of the lesion increased, the possibility of SA significantly decreased, with a cut-off value of 10 mm. When the size exceeded 20 mm, it tended to stabilize. Nevertheless, these two sets of variables exhibited a non-linear relationship with the probability of SA (*P*<0.001) (**Figures 2A, B**). Premenopausal asymptomatic women with unpalpable nodules, distinct margins, homogeneous internal echo, a blood flow level of 0-I, and an RI of <0.70 were all predictive factors of SA.

**Figure 3:**
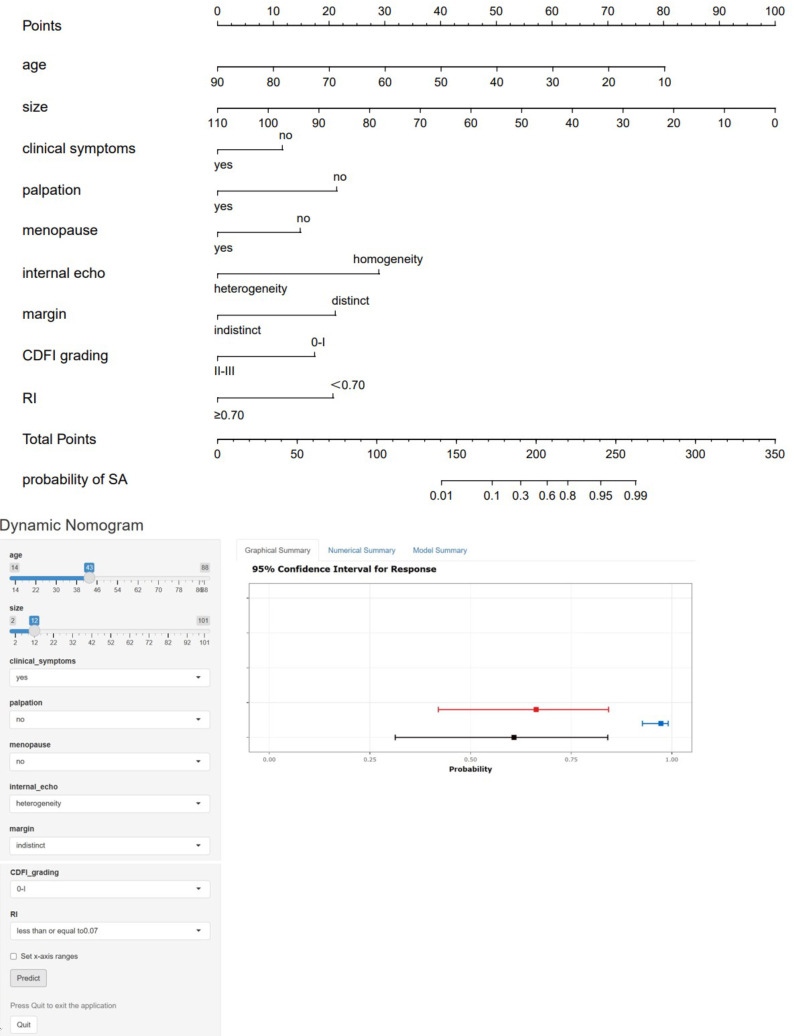
Static and dynamic nomogram model. The nomogram was constructed based on logistic regression analysis results of ultrasound features to predict the diagnosis of SA, and the dynamic nomogram is available at http://saprediction.shinyapps.io/SAdynamic. The model consisted of age (years), size (mm), menopausal status, clinical symptoms, palpation, margin, internal echo, CDFI grading, and RI.

**Figure 4:**
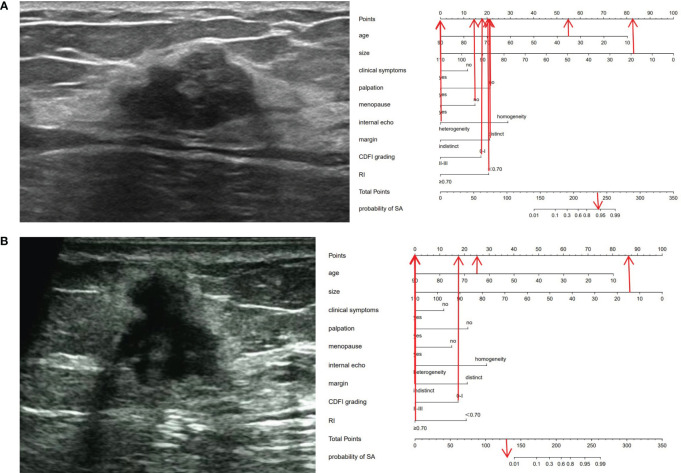
Ultrasonic cases and their nomograms. **(A)** A 35-year-old (55 points) female patient, who was premenopausal (15 points), sought medical attention due to breast pain (0 points) and had no evident mass on palpation (22 points). Ultrasound examination revealed a 19 × 17 × 13 mm nodule (82 points) in the left breast, with distinct margins (22 points), heterogeneous (0 points), with a CDFI level of 0-I (18 points), an RI of 0.59 (20 points), and was categorized as BI-RADS 4a. The total score was 234 (55 + 15 + 0+22 + 82 + 22 + 0+18 + 20 = 234). The probability of SA for this nodule was greater than 0.9. Histopathological results: SA. **(B)** A 65-year-old (25 points) female patient, who was menopausal (0 points), sought medical attention owing to breast pain and a conscious breast nodule (0 points), and the nodule was palpable (0 points). Ultrasound examination delineated a 14×13 ×13 mm nodule (86 points) in the left breast, with indistinct margins (0 points), heterogeneous (0 points), a CDFI level of 0-I (18 points), an RI of 0.73 (0 points), and was classified as BI-RADS 5. The total score was 129 points (25 + 0 + 0+0 + 86 + 0+0 + 18 + 0 = 129 points). The probability of SA for this nodule was less than 0.01. Histopathological results: IDC.

These statistically significant indicators were used to construct a static nomogram and an online dynamic nomogram (**Figure 3**) (http://saprediction.shinyapps.io/SAdynamic) to predict the probability of SA. According to the nomogram, the risk of SA for any nodule could be determined by the sum of scores obtained from various ultrasound indicators (**Figures 4A, B**).

### 3.4 Evaluation of nomogram

**Figure 5:**
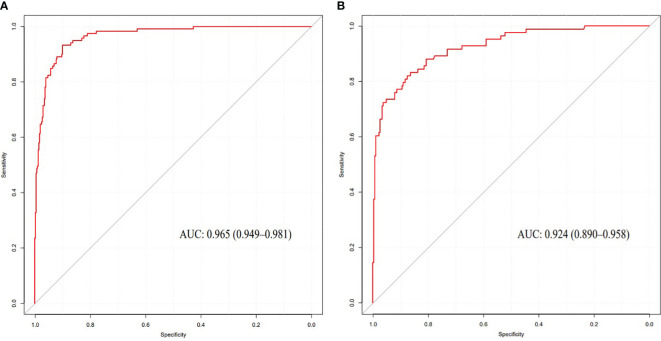
ROC curve of the training set **(A)** and validation set **(B)**.

**Figure 6:**
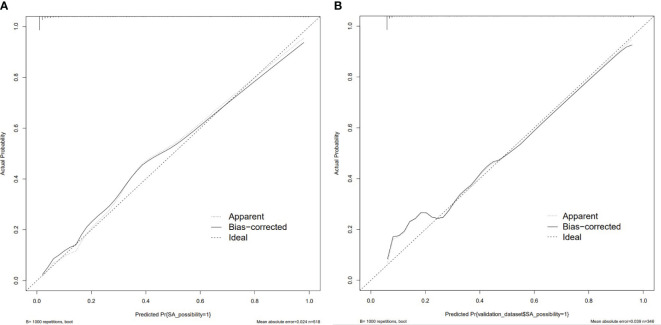
Calibration curve of the training set **(A)** and validation set **(B)**. The calibration curve represents the relationship between the predicted probability (x-axis) and the actual probability (y-axis) of SA, the dashed line on the diagonal portrays the predicted probability=actual probability, and the solid line represents the calibration curve of the nomogram. The curves of the training and validation set were close to the dashed line, indicating high calibration accuracy.

**Figure 7:**
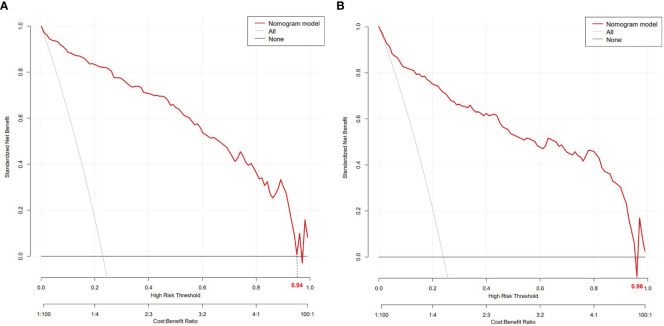
DCA of the training set **(A)** and validation set **(B)**. In both the training set (threshold 0-0.94) and the validation set (threshold 0-0.96), the nomogram exhibited clinical benefits.

The nomogram model had high predictive performance, with an AUC of 0.965 (95% CI: 0.949-0.981) in the training set and 0.924 (95% CI: 0.890-0.958) in the validation set (**Figure 5**). The calibration curves of both the training and validation sets exposed that the model had satisfactory calibration (**Figure 6**). DCA demonstrated that the model possessed clinical benefits (**Figure 7**).

## 4 Discussion

Our study established that nearly 50% of SA nodules present with malignant signs and are BI-RADS 4a or above, related to its pathological characteristics. Compared with IDC, the size of SA nodules is smaller, and SA does not clinically manifest symptoms, thereby complicating palpation and preoperative ultrasonic diagnosis. Therefore, this ultrasound-based comparative study between SA and IDC enabled the construction of a nomogram to assist ultrasound physicians in not only further analyzing the value of various ultrasonic features in SA diagnosis but also calculating the total score on the nomogram to predict the risk of SA (17–19). Our research indicated that age, nodule size, menopausal status, presence of clinical symptoms, palpability of lesions, margins, internal echo, CDFI grading, and RI could be used as predictive factors for distinguishing between SA and IDC. Herein, age and nodule size were identified as the strongest predictive factors. Furthermore, the nomogram model exhibited favorable predictive performance (AUC>0.9), calibration, and clinical application value, in both the training and validation sets.

According to previous studies, clinical nomograms have been applied to breast diseases in numerous aspects, such as predicting breast cancer, assessing the diagnostic performance of imaging methods in breast diseases (18–21), predicting axillary lymph node metastasis of breast cancer (22), and predicting the recurrence rate and risk of breast cancer (23), etc. In the ultrasonic differentiation between SA and malignant tumors, the findings of Liang’s study were consistent with ours (17). Nevertheless, our sample size is larger than their study. When determining indicators to be included in the logistic analysis and nomogram construction, in addition to the patient’s age, nodule size, and ultrasound indicators involved in BI-RADS, the significance of the patient’s symptoms and physical examination results should be considered in disease differentiation to better combine ultrasound diagnosis with clinical manifestations. This model can serve as a supplement and auxiliary to BI-RADS grading and be applied for clinical diagnosis. Moreover, this study developed an online dynamic nomogram, providing a more convenient tool for clinical doctors.

Malignant lesions consistently exhibit infiltrative growth, with a higher growth rate than benign lesions (24). In our study, the proportion of SA lesions with a size exceeding 20 mm was lower than 20%, whereas the size of nearly 50% of IDC lesions exceeded 20 mm, and merely less than 10% of lesions had a size smaller than 10 mm, which could be attributed to the growth pattern of the lesions. In addition, a correlation was discovered between lesion size and SA through RCS curves. In terms of size alone, the smaller the size, the greater the likelihood of SA, with a cut-off value of 10 mm, and this relationship tended to stabilize above 20 mm, which has not been reported in the literature so far. Moreover, age is an independent risk factor for breast cancer that has been confirmed at the molecular level (25). In the current study, age was also a predictive factor for distinguishing SA from IDC. The younger the patient, the greater the likelihood of SA, which is in line with the observations of previous studies (17). Notably, the cut-off value was 36 years old in our study, and the relationship between age and SA tended to stabilize after 55 years old. In this study, the average age of patients in the SA group was significantly lower than those in the IDC group (40.0 ± 12.3 vs. 53.6 ± 12.2, 42.2 ± 10.8 vs. 52.3 ± 11.4), with over 80% of SA patients in the premenopausal state, which was significantly different from IDC patients. Besides, the mean age of SA patients was marginally lower than previously reported (43.2-50.0 years) (4, 6, 10, 26–28), and this difference may be explained by the current emphasis on breast ultrasound examination in young patients. As is well documented, SA is related to the risk of breast cancer. Younger trend of breast cancer may lead to younger SA (29). However, this hypothesis warrants additional investigations with larger sample data.

According to our study, typical ultrasonic features of IDC were an irregular hypoechoic silhouette accompanied by microcalcification, visible echogenic rim, and abundant blood supply. On the other hand, SA lesions were predominantly hypoechoic, more commonly manifesting as lesions with distinct margins, regular shapes, homogeneous internal echoes, lateral growth, absence of calcification, lack of blood supply, and low RI, which is in agreement with the results of previous studies (4). Among them, margin, internal echo, CDFI grading, and RI are key features for distinguishing between SA and IDC.

The complex features of SA, such as irregular shape, posterior shadow, and microcalcification, are significantly distinct from other common benign lesions, leading to an overestimation of the level of these lesions in BI-RADS (17, 28, 30). Unlike the invasive growth of malignant tumors, the irregular shape of SA is principally related to interstitial sclerosis (30). Practically 50% of IDC lesions exhibit posterior shadow, which is consistent with our study results and is associated with the proliferation of connective tissue adjacent to malignant tumors (31). The pathological basis for the difference in the posterior shadow of SA is different from that of IDC. The shadow of SA is caused by the fibrous components in the lesion, and fibrocystic changes can also lead to the appearance of cystic components within the lesion, presenting a cystic, solid appearance (28). The morphology and distribution of microcalcifications can aid in predicting the risk of breast malignant tumors. In terms of morphology, benign lesions are more susceptible to exhibiting amorphous or coarse calcifications, whereas microcalcifications are more prone to malignancy. In terms of distribution, lesions with regional distribution are more likely to be benign; in contrast, lesions with segmental or linear distribution tend to be malignant. Mammography is highly sensitive to microcalcification (32, 33). Indeed, microcalcification is an essential manifestation of mammography. Earlier studies have reported that 20-40% of SA lesions exhibit microcalcification, and the morphology and distribution of microcalcification are comparable to malignant tumors (5, 10, 19). In a breast ultrasound examination, SA lesions with microcalcification are more susceptible to being classified by ultrasound physicians as BI-RADS level 4 or higher. In our study, microcalcification could be visualized in only about 10% of SA lesions, which is not only related to the low sensitivity of ultrasound to calcification (10) but also to the physician’s perception of microcalcification during retrospective image analysis. The combination of mammography and ultrasound can enhance the recognition of malignant microcalcifications. However, the former has a more individualized predictive value for dense breasts (32, 33). Therefore, in clinical practice, mammography is recommended for suspicious SA lesions accompanied by microcalcifications. If necessary, a needle localization biopsy may be performed (34).

This study has several limitations that should be considered. To begin, this was a retrospective study that is inherently subjected to bias. Secondly, the ultrasonic features involved in this study were all two-dimensional and color Doppler features. In future research, elastic imaging and contrast-enhanced ultrasound should be included to examine the role of multimodal ultrasound in the differentiation of SA and IDC. Thirdly, the nomogram model constructed in this study was validated by internal data but lacked external validation. Thus, the effectiveness of the model should be further evaluated.

## 5 Conclusion

A diagnosis of SA should be considered in asymptomatic young women, especially those younger than 36 years of age, who present with small-size lesions (especially less than 10 mm) with distinct margins, homogeneous internal echo, and lack of blood supply. The nomogram model can serve as a supplement and auxiliary for BI-RADS and can be used to distinguish SA from IDC. Overall, the dynamic nomogram model provides a more convenient tool for clinicians.

## Author contributions

YL: Data curation, Methodology, Writing – original draft. X-LW: Data curation, Writing – review & editing. K-KP: Data curation, Formal Analysis, Writing – review & editing. P-JN: Data curation, Writing – review & editing. MW: Supervision, Writing – review & editing. JX: Data curation, Formal Analysis, Writing – review & editing. L-LZ: Data curation, Writing – review & editing. F-XZ: Data curation, Supervision, Writing – review & editing.
